# Millipede accident with unusual dermatological lesion^☆^^☆☆^

**DOI:** 10.1016/j.abd.2019.10.003

**Published:** 2019-11-06

**Authors:** Silmara Navarro Pennini, Paula Frassinetti Bessa Rebello, Maria das Graças Vale Barbosa Guerra, Sinésio Talhari

**Affiliations:** a Post-graduate Program in Tropical Medicine, Fundação de Medicina Tropical Dr. Heitor Vieira Dourado e Universidade do Estado do Amazonas, Manaus, AM, Brazil; b Fundação de Dermatologia Tropical e Venereologia Alfredo da Matta, Manaus, AM, Brazil

Dear Editor;

A 32-year-old male patient reported that, upon waking, he noticed injuries to his right leg, with local burning sensation and no other symptoms, and that he saw a millipede on the bed (Figure 1, Figure 2, Figure 3). He went to the emergency room, where he was treated with antihistamines. Due to a lack of improvement, he sought a dermatologist. At the physical examination, the patient presented three spiral-shaped erythematous brownish spots, measuring approximately 3 cm each, located on the anterolateral surface of the right thigh. Clobetasol 0.05% ointment was prescribed, leading to an improvement of the burning sensation of the lesions.

**Figure 1:**
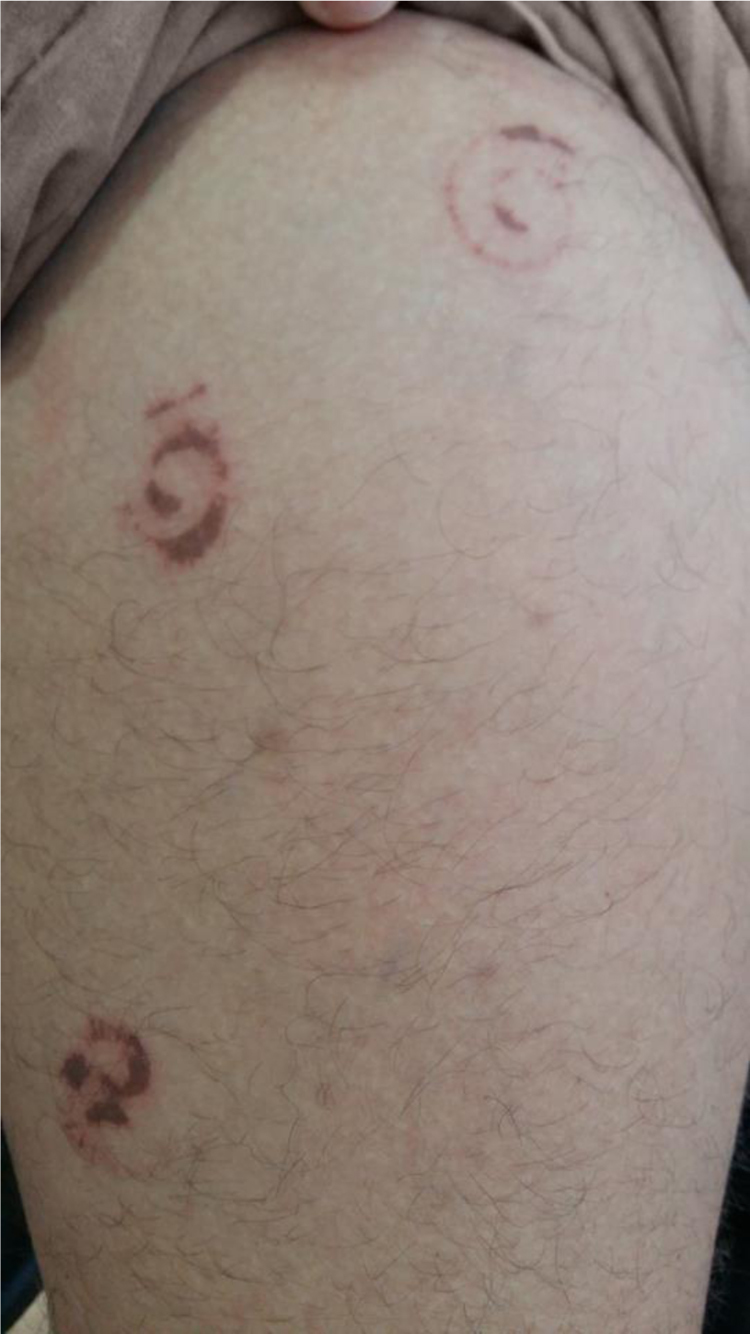
Spiral-shaped erythematous-brown spot on the right thigh.

**Figure 2:**
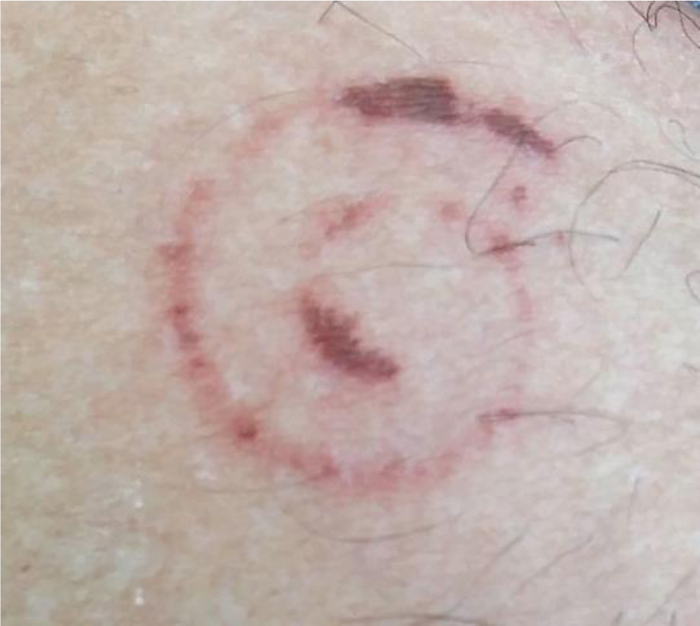
Detail of the lesion.

**Figure 3:**
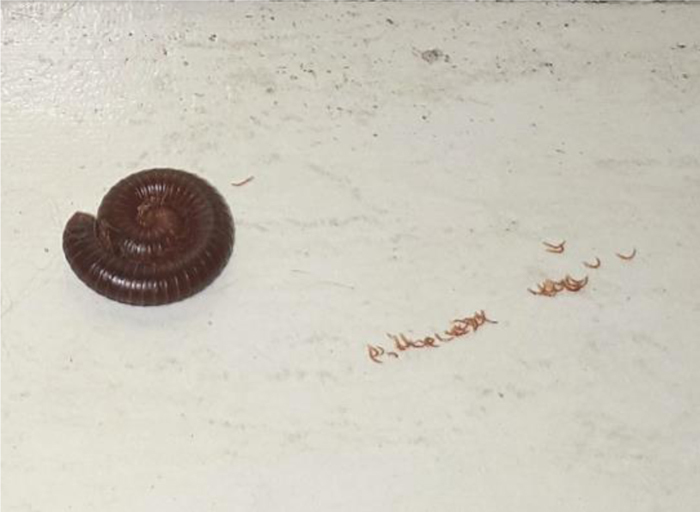
Millipede after curling.

Millipedes are animals of the phylum Arthropoda, the group with the largest number of invertebrate species. A member of the Diplopoda class, they have a cylindrical, segmented, rigid exoskeleton and two pairs of appendages or limbs (myriads) articulated in each segment, which move symmetrically and slowly, unlike centipedes (Chilopoda), which have only one pair of limbs per segment for support and thrust, giving them faster movement.^1^

Millipedes are nocturnal animals that inhabit dark and humid places, and have two defense mechanisms: spiral curling (with the head in the center), providing greater resistance to the exoskeleton, and the discharge of an irritating secretion, which flows from glands on the lateral portion of each body segment when the animal is under threat or being crushed. The secretion can also be ejected from a distance.^2^, ^3^

The species related to human accidents in Brazil, *Rhinocricus padbergi* (family Rhinocricidae), is a member of the Spirobolida order, whose secretion is mainly composed of benzoquinone (2-methyl-1,4-benzoquinone), a highly irritating compound.^2^

These are mostly harmless animals; however, when defending themselves, they can excrete toxins that cause irritating and pigmenting chemical reactions in the skin.^3^, ^4^ Accidents with children and adults usually occur when they are unconscious, lying on the floor, or during contact with clothes and shoes, especially during the rainy season, when millipedes invade urban areas and houses in search of shelter in a dark place.^5^ Almost immediately after contact, there is numbness and a burning sensation on the skin.^4^ The affected site becomes erythematous, with initially brownish-yellow pigmentation, darkening after 24 h and turning reddish-brown to black, with a cyanotic appearance, a coloration that may persist for several months.^3^ Depending on the amount of secretion and exposure time, the pigmented lesion may dry out and peel in approximately seven days, or there may be blistering that, upon rupture, leaves the surface eroded.^2^

Most case reports describe pigmented lesions without a definite shape resulting from crushing the millipede. The present case is particularly interesting because the lesions reproduced the body shape of the millipede, in the defensive position, as an impression of the animal on the skin, mirroring the position of the secretory glands.

## Financial support

None declared.

## Authors’ contribution

Silmara Navarro Pennini: Conception and planning of the study; preparation and writing of the manuscript; intellectual participation in propaedeutic and/or therapeutic conduct of the studied case.

Paula Frassinetti Bessa Rebello: Preparation and writing of the manuscript; intellectual participation in propaedeutic and/or therapeutic conduct of studied cases; critical review of the literature.

Maria das Graças Vale Barbosa Guerra: Preparation and writing of the manuscript; critical review of the literature; critical revision of the manuscript.

Sinésio Talhari: Approval of the final version of the manuscript; participation in the study design and planning; critical revision of the manuscript.

## Conflicts of interest

None declared.
